# Pycnogenol^®^ Supplementation Attenuates Memory Deficits and Protects Hippocampal CA1 Pyramidal Neurons via Antioxidative Role in a Gerbil Model of Transient Forebrain Ischemia

**DOI:** 10.3390/nu12082477

**Published:** 2020-08-17

**Authors:** Bora Kim, Tae-Kyeong Lee, Cheol Woo Park, Dae Won Kim, Ji Hyeon Ahn, Hyejin Sim, Jae-Chul Lee, Go Eun Yang, Jong Dai Kim, Myoung Cheol Shin, Jun Hwi Cho, Sungwoo Ryoo, Young-Myeong Kim, Moo-Ho Won, Joon Ha Park

**Affiliations:** 1 Department of Neurobiology, School of Medicine, Kangwon National University, Chuncheon, Gangwon 24341, Korea; nbrkim17@gmail.com (B.K.); flfhflfh@naver.com (C.W.P.); jh-ahn@hallym.ac.kr (J.H.A.); janny20@kangwon.ac.kr (H.S.); anajclee@kangwon.ac.kr (J.-C.L.); 2 Department of Biomedical Science and Research Institute for Bioscience and Biotechnology, Hallym University, Chuncheon, Gangwon 24252, Korea; tk-lee@hallym.ac.kr; 3 Department of Biochemistry and Molecular Biology and Research Institute of Oral Sciences, College of Dentistry, Gangnung-Wonju National University, Gangneung, Gangwon 25457, Korea; kimdw@gwnu.ac.kr; 4 Department of Radiology, Kangwon National University Hospital, Chuncheon, Gangwon 24289, Korea; yangke@kangwon.ac.kr; 5 Division of Food Biotechnology, School of Biotechnology, Kangwon National University, Chuncheon, Gangwon 24341, Korea; jongdai@kangwon.ac.kr; 6 Department of Emergency Medicine, and Institute of Medical Sciences, Kangwon National University Hospital, School of Medicine, Kangwon National University, Chuncheon, Gangwon 24289, Korea; dr10126@naver.com (M.C.S.); cjhemd@kangwon.ac.kr (J.H.C.); 7 Department of Biological Sciences, Kangwon National University, Chuncheon, Gangwon 24341, Korea; ryoosw08@kangwon.ac.kr; 8 Department of Molecular and Cellular Biochemistry, School of Medicine, Kangwon National University, Chuncheon, Gangwon 24341, Korea; ymkim@kangwon.ac.kr; 9 Department of Anatomy, College of Korean Medicine, Dongguk University, Gyeongju, Gyeongbuk 38066, Korea

**Keywords:** antioxidation, antioxidant enzymes, free radicals, ischemia-reperfusion injury, neuroprotection, oxidative stress

## Abstract

Pycnogenol^®^ (an extract of the bark of French maritime pine tree) is used for dietary supplement and known to have excellent antioxidative efficacy. However, there are few reports on neuroprotective effect of Pycnogenol^®^ supplementation and its mechanisms against ischemic injury following transient forebrain ischemia (TFI) in gerbils. Now, we examined neuroprotective effect and its mechanisms of Pycnogenol^®^ in the gerbils with 5-min TFI, which evokes a significant death (loss) of pyramidal cells located in the cornu ammonis (CA1) region of gerbil hippocampus from 4–5 days post-TFI. Gerbils were pretreated with 30, 40, and 50 mg/kg of Pycnogenol^®^ once a day for 7 days before TFI surgery. Treatment with 50 mg/kg, not 30 or 40 mg/kg, of Pycnogenol^®^ potently protected learning and memory, as well as CA1 pyramidal cells, from ischemic injury. Treatment with 50 mg/kg Pycnogenol^®^ significantly enhanced immunoreactivity of antioxidant enzymes (superoxide dismutases and catalase) in the pyramidal cells before and after TFI induction. Furthermore, the treatment significantly reduced the generation of superoxide anion, ribonucleic acid oxidation and lipid peroxidation in the pyramidal cells. Moreover, interestingly, its neuroprotective effect was abolished by administration of sodium azide (a potent inhibitor of SODs and catalase activities). Taken together, current results clearly indicate that Pycnogenol^®^ supplementation can prevent neurons from ischemic stroke through its potent antioxidative role.

## 1. Introduction

Transient brain ischemia occurs following transient block or reduction of blood flow to brain tissue, and it results in irreversible injury to a large part of the brain, which can cause neurological deficits including cognitive and motor impairments [1,2]. Even though mechanisms underlying transient ischemia-induced brain injury remain unclear, oxidative stress following abnormal production of reactive oxygen species (ROS) is a major factor in pathophysiology of brain ischemia, and antioxidants may represent promising targets for deriving neuroprotection against ischemic brain injury [3,4].

Plant extracts contain a variety of potential effector components which have been recognized as multitarget therapeutic agents because of their broad spectrum of pharmacological actions [5,6,7]. Pine bark is known as a rich source of natural polyphenols, including proanthocyanidins which have high antioxidant capacity [8,9], and it has been utilized in traditional folk medicine for broad health issues [10]. Pine bark extract is utilized in fields of nutrition, cosmetics, and medicine [11,12].

Pycnogenol^®^ (PYC), which is produced by Horphag Research Co. Ltd. (Geneva, Switzerland), is an extract of natural plant originating from the bark of French maritime pine tree (*Pinus pinaster*). It has been reported that PYC plays effects against some experimental neurological disorders. For example, PYC protects neuronal cells from apoptosis induced by amyloid β peptide [13], neuroinflammation induced by 1-methyl-4-phenyl-1, 2, 3, 6-tetrahydropyridine (MPTP), and neurodegeneration [14]. In addition, it improves spatial memory deficit in mice with Alzheimer’s disease [15]. In particular, Ozoner et al. (2019) recently reported that PYC protected inflammatory and oxidative injury in rat brains following transient focal brain ischemia that was induced by ligation of middle cerebral artery [16].

To the best of our knowledge, few research studies on neuroprotective effect of PYC against transient forebrain ischemia (TFI) and its protective mechanisms have been reported. TFI can be induced in gerbils by occlusion of common carotid arteries (OCCA) in gerbils and evokes death (loss) of pyramidal cells, which are principal neurons in the cornu ammonis (CA1) region of gerbil hippocampus, and memory deficits [17,18,19,20]. Therefore, the aim of this research was to examine neuroprotective effect of PYC and its protective mechanisms in gerbils with TFI.

## 2. Materials and Methods

### 2.1. Experimental Animals

Male gerbils (age, six months; body weight, 72–78 g) were provided from the Experimental Animal Center located in Kangwon National University (Kangwon, Korea). The obtained gerbils had free access to water and feed and cared for under optimum status with appropriate temperature (23 °C) and humidity (60%), which was given cycle of 12 h light and 12 h darkness. The experimental protocol of this research was approved (approval no., KW-200113-1) at 18 February 2020 by Institutional Animal Care and Use Committee (AICUC) of Kangwon National University and adhered to the guidelines [21].

### 2.2. PYC Preparation and Characteristics

PYC was purchased from Horphag Research Co., Ltd. (Geneva, Switzerland). PYC dominantly contains phenolic compounds [22,23] and, among them, standardized procyanidins which are members of proanthocyanidin class of flavonoids mainly occupies (about 65~75%). Besides it contains bioflavonoids, organic acids and a trace part of inorganic ions. As presented in the Table 1, the composition of PYC was listed in detail [12,24]. The source of PYC is the bark of maritime pine which is a single species of pine trees exclusively grown in southwest of France (Les Landes de Gascogne forest).

### 2.3. Experimental Groups and PYC Supplementation

In this study, eight groups were used as follows: (1) vehicle/sham group (*n* = 13) received vehicle and sham operation; (2) vehicle/TFI group (*n* = 26) received vehicle and TFI operation; (3), (4) and (5) 30, 40 and 50 mg/kg PYC/sham groups (*n* = 7, 7 and 13, respectively) which were administered with 30, 40 and 50 mg/kg of PYC, respectively, and given sham operation; (6), (7) and (8) 30, 40 and 50 mg/kg PYC/TFI groups (*n* = 7, 7 and 26, respectively) which were treated with 30, 40 and 50 mg/kg of PYC and undergone TFI operation. PYC was dissolved in saline (0.85% sodium chloride *w*/*v*) as a vehicle. PYC or vehicle was intraperitoneally administered once a day for 7 days before ischemic surgery.

### 2.4. TFI Induction

Surgical procedure for induction of TFI was performed as described in our previous paper [25]. Shortly, the gerbils used for TFI were anesthetized with a mixture of 2.5% of isoflurane (Hana Pharm. Co., Kyeonggi-Do, Korea) in 33% of oxygen (O^2^) and 67% of nitrous oxide (N_2_O) using an inhaler. Under anesthesia, both common carotid arteries, which supply blood to brains, were isolated from the carotid sheath in the neck and ligated by clips for 5 min. Immediately, ischemia (no blood supply to the brains) was confirmed by observing the stop of blood circulation in the central artery located in the retinae with an ophthalmoscope. After the confirmation, the clips were removed, blood circulation was confirmed in the retinae, and the incised tissue in the neck was sutured. During the surgical procedure, body temperature of the gerbils was controlled at 37 ± 0.5 ℃ using a rectal temperature probe (TR-100) from Fine Science Tools (Foster City, CA, USA). In addition, the temperature was controlled until the gerbils renewed their strength. Sham gerbils received the same TFI surgical procedure without occlusion of the carotid arteries.

### 2.5. Tests of Learning and Memory

#### 2.5.1. Passive Avoidance Test (PAT)

To investigate learning and memory function following TFI, PAT was performed at 5 days after TFI. In short, as previously described [19,26], PAT test was done by using Gemini Avoidance System (GEM 392) (San Diego Instruments, San Diego, CA, USA). This system was made up of light and dark compartments separated by a vertical gate. Training for the PAT was performed twice a day for 3 days before TFI. In detail, the gerbils were permitted to freely explore the two compartments for 2 min. The vertical gate was closed followed by giving foot-shock (0.5 mA) for 3 s when the gerbil went into the dark compartment. The substantive test was carried out at 1 day before and 5 days after TFI. Namely, each gerbil was placed onto the light compartment, as the vertical gate was opened. The latency time in each gerbil was recorded till the gerbil entered the dark room. In this experiment, the highest latency time to keep in the light room was designated as 180 s.

#### 2.5.2. 8-Arm Radial Maze Test

To examine working and spatial memory function in each group, 8-arm radial maze test was performed five days after TFI according to a method with some modifications by Liu et al. (2014) [27]. In brief, we used an apparatus for the maze consisted of a central platform (20 cm diameter) and radially extended 8 arms (6 cm width, 6 cm height, 25 cm length). In order to make the gerbils habituated to the maze and pellet feeds, the gerbils were pre-trained twice/day for 3 days before TFI. The actual trials were conducted once a day for last 3 days (day 3, 4 and 5) after TFI. Namely, a feed was located at the end of each arm, each gerbil was put onto the central platform, and the trial was recorded when the gerbil ate the feed. Finally, numbers of the errors were recorded when the gerbil went into an arm which the gerbil had already went before.

### 2.6. Preparation of Tissue Sections

As described in our published paper [28], tissue sections containing the hippocampus were prepared at 5 days after sham operation, and at 2 and 5 days after TFI operation, respectively. In brief, gerbils (*n* = 7 at each point in time) in each group were given deep anesthesia by intraperitoneal injection of 1.5 g/kg urethane (Sigma-Aldrich, St. Louis, MO, USA). Under anesthesia, their brains were rinsed by perfusion of 0.1 M PBS (pH 7.4) through the ascending aorta followed by 4% paraformaldehyde solution (in 0.1 M PB, pH 7.4) to fixed the brains. The fixed brains were taken out from the heads and stored in the same fixative. Five hours later, the brains were stored in 30% sucrose solution for 12 h to prevent the brain from cryosection. The cryoprotected brains were sectioned into coronal planes of 30 μm of thickness in a cryostat.

### 2.7. Histochemistry with Cresyl Violet (CV)

As described in our published paper [1], CV staining (a method to detect Nissl’s body) was done in order to assess protective effects of PYC on hippocampal neuronal damage induced by TFI. Briefly, the tissue sections were put onto microscopy slides coated with gelatin. These brain sections were stained in solution of 1.0% CV acetate (Sigma-Aldrich, St. Louis, MO, USA) for 20 min at room temperature. Subsequently the stained sections were dehydrated in baths of serial ethanol. These dehydrated sections were covered for permanent slides using Canada balsam (Kanto, Tokyo, Japan), a clear transparent film.

To examine neuronal damage or survival, five brain sections were chosen with 120 μm interval between anteroposterior −1.4 and −2.2 mm of the brain drawn in the gerbil brain atlas [29]. As previously described [30], change of CV positive (CV^+^) cells were examined using light microscope (BX53) from Olympus (Deutschland GmbH, Hamburg, Germany).

### 2.8. Histofluorescence with Fluoro-Jade B (F-J B)

Histofluorescence with F-J B, a marker for cellular or neuronal degeneration, was performed to examine neuroprotective effects of PYC on hippocampal neuronal damage/death following TFI. Briefly, as described in our previous paper [31], the prepared sections were incubated in 1% sodium hydroxide solution, transferred to 0.06% potassium permanganate solution followed by 0.004% F-J B (Histochem, Jefferson, AR, USA) solution. These sections were washed with saline and put on a slide warmer (50 ± 0.5 °C) for reaction of F-J B.

Damage/death of cells in gerbil hippocampus was quantitatively analyzed as follows. Five sections were chosen as described above. F-J B positive (F-J B^+^) cells was counted according to method by Lee et al. [30]. Briefly, digital images of the F-J B^+^ cells were captured using epifluorescence microscope (BX53) from Olympus (Deutschland GmbH, Hamburg, Germany) equipped with blue (450–490 nm) excitation light. The F-J B^+^ cells were counted in a 100 × 100 μm^2^ at the middle of the CA1 region. Finally, the count of F-J B^+^ cells was done by averaging total numbers of the F-J B^+^ cells with image analyzing system (Optimas 6.5) from CyberMetrics (Scottsdale, AZ, USA).

### 2.9. Immunohistochemical Stainings

Immunohistochemical stainings were performed to investigate with following primary antibodies (1) neuronal damage/death with neuronal nuclear antigen (NeuN) as a marker for neuron, (2) oxidative stress with 8-hydroxyguanine (8OHG) as a marker of oxidative nuclear stress and 4-hydroxy-2-nonenal (4HNE) as a marker for lipid peroxidation, (3) endogenous antioxidants with Cu, Zn-superoxide dismutase (SOD1), Mn-superoxide dismutase (SOD2), and catalase (CAT). According to method by Le et al. [30] with some modifications, the sections were reacted in each diluted primary antibody solution: mouse anti-NeuN (diluted 1:1000, Chemicon, Temecula, CA, USA), goat anti-8OHG (1:500, ThermoFisher Scientific, Waltham, MA, USA), mouse anti-4HNE (1:1000, Alexis Biochemicals, San Diego, CA, USA), sheep anti-SOD1 (diluted 1:1000, Calbiochem, La Jolla, CA, USA), sheep anti-SOD2 (diluted 1:1000, Calbiochem), and rabbit anti-CAT (diluted 1:1000, Abfrontier, Seoul, Korea). Continuously, these reacted sections were incubated in secondary antibody solution: biotinylated donkey anti-mouse, goat, sheep or rabbit IgG (diluted 1:250, Vector, Burlingame, CA, USA) and avidin-biotin complex (diluted 1:300, Vector, Burlingame, CA, USA). Continually, these immunoreacted sections were reacted with solution of 3, 3′-diaminobenzidine tetrahydrochloride (DAB) (Sigma-Aldrich, St. Louis, MO, USA) to be vitalized. 

Negative control tests were performed, to examine the specificity of each immunostaining, with preimmune serum. As a result, no immunostained structures were shown in the tested sections (data not shown).

For quantitative analysis of the immunostained structures, five sections were selected as described above. NeuN^+^ neurons were analyzed according to method by Lee et al. [30]. Briefly, digital images of the NeuN^+^ cells were captured in a 100 × 100 μm^2^ at the middle of the CA1 region with a light microscope. The count of NeuN^+^ neurons was done by averaging total numbers of NeuN^+^ neurons by using the above-mentioned image analyzing system.

For quantitative analysis of the immunoreactivity in 8OHG^+^, 4HNE^+^, SOD1^+^, SOD2^+^, and CAT^+^ structures, we carried out it according to method by Kim et al. [28]. Shortly, each digital image was captured like the method mentioned above. The immunoreactivity of each structure was evaluated as relative immunoreactivity (RI) as % by using Adobe Photoshop (version 8.0) from San Jose (San Jose, CA, USA) and NIH Image software (version 1.59) (NIH, Bethesda, MD, USA).

### 2.10. Dihydroethidium (DHE) Histofluorescence

We used DHE that was purchased from Sigma-Aldrich (St. Louis, MO, USA) to evaluate in situ production of superoxide anion. The DHE is an oxidative fluorescent dye. DHE histofluorescence was done according to method by Kim et al. [28] with some modifications. In brief, firstly, the sections were equilibrated in Krebs-HEPES buffer (pH 7.4), which consisted of 130 mM NaCl, 2 mM CaCl2, 5.6 mM KCl, 0.24 mM MgCl2, 11 mM glucose, and 8.3 mM HEPES, for 30 min at 37 °C and immediately applied with fresh buffer containing DHE (10 μmol/L). Finally, these sections were covered with coverglasses and incubated in humidified dark chambers for 2 h at 37 °C. 

The intensity of DHE histofluorescence was evaluated measured as described in a paper by Lee et al. [1]. Briefly, digital image of DHE histofluorescence was captured from the area of interest with epifluorescence microscope (BX53; Olympus, Tokyo, Japan) like the method described above. The histofluorescence intensity of DHE was analyzed as % with Image-pro Plus 6.0 software (Media cybernetics Inc., MD, USA).

### 2.11. Treatment of Sodium Azide

To elucidate whether PYC treatment contributed to increasing endogenous antioxidant enzymes in the hippocampus, we used sodium azide (SA) (Sigma-Aldrich, St. Louis, MO, USA) which is a potent inhibitor of SODs and CAT activities [32]. We prepared PYC/SA/sham group (*n* = 26) and PYC/SA/TFI group (*n* = 26) at 2 days (*n* = 13) and 5 days (*n* = 13) after TFI. Twenty mg/kg of SA was dissolved with saline and intraperitoneal injection was done at 10 min after PYC treatment. The SA dose was chosen according to method by Somade et al. [33].

### 2.12. Statistical Analysis

Data obtained in this study are presented as the means ± standard error of the mean (SEM). All of the statistical analyses were performed with GraphPad Prism (version 5.0) (GraphPad Software, La Jolla, CA, USA). Differences of the means among all of the groups were analyzed by two-way analysis of variance (ANOVA) with a post hoc Bonferroni’s multiple comparison test to elucidate TFI-mediated differences between all groups. *p* < 0.05 was used for statistical significance.

## 3. Results

### 3.1. Attenuation of Learning and Memory Deficits

#### 3.1.1. PAT

**Figure 1:**
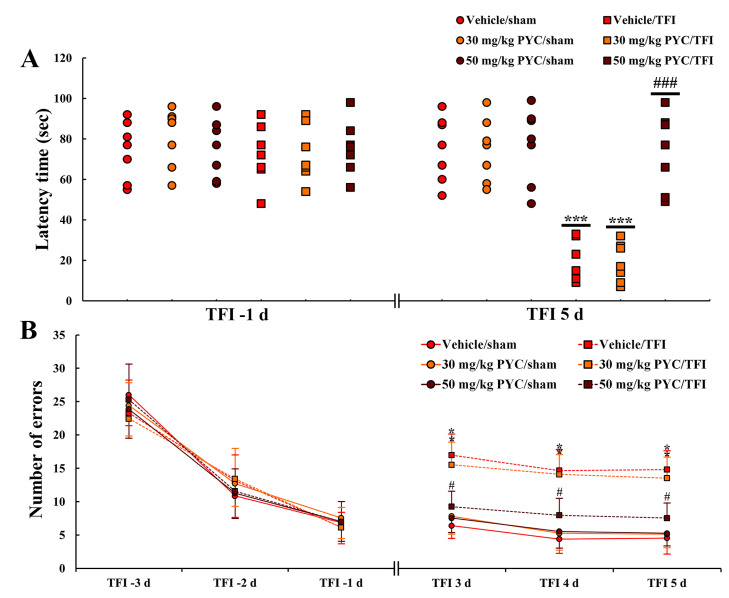
(**A**) Latency time in Passive Avoidance Test (PAT) in the vehicle/sham, vehicle/transient forebrain ischemia (TFI), PYC (30, 50 mg/kg)/sham and PYC/TFI groups at 5 days after TFI. (**B**) Number of errors in 8-arm radial maze test in the vehicle/sham, vehicle/TFI, PYC (30, 50 mg/kg)/sham and PYC/TFI groups at 3, 4, and 5 days after TFI. The bars indicate the means ± SEM (*n* = 7/group, *** *p* < 0.001 vs. each sham group, ^###^ *p* < 0.001 vs. vehicle/TFI group, * *p* < 0.05 vs. corresponding time-point vehicle/sham group, ^#^ *p* < 0.05 vs. vehicle/TFI group).

Latency times at 1 day before TFI were not significantly different among all groups (Figure 1A). In all sham groups (vehicle/sham, and 30, 40 and 50 mg/kg PYC/sham), there were no significant differences in latency time at 5 days after TFI (Figure 1A). On the other hand, in the vehicle/TFI, 30 and 40 mg/kg PYC/TFI groups, the latency time was shortened in comparison with that in the vehicle/sham group (Figure 1A). However, the latency time in the 50 mg/kg PYC/TFI group was not significantly different from that in the vehicle/sham group (Figure 1A): this finding means that PYC treatment protects learning and memory function against ischemic insult.

#### 3.1.2. 8-Arm Radial Maze Test

Patterns in numbers of errors before TFI induction were similar (Figure 1B). In contrast, in all of the sham groups (vehicle/sham, and 30, 40 and 50 mg/kg PYC/sham) groups, the numbers of errors were similar among these groups at 3, 4 and 5 days after TFI (Figure 1B). In contrast, the numbers of errors in the vehicle/TFI, 30 and 40 mg/kg PYC/TFI groups, were significantly high in comparison to those in the vehicle/sham group (Figure 1B). However, in the 50 mg/kg PYC/TFI group, the numbers of errors were remarkably low compared to the other TFI groups (Figure 1B): this finding means that PYC treatment attenuates TFI-induced spatial memory deficit.

### 3.2. Protection of CA1 Pyramidal Cells

#### 3.2.1. CV^+^ Cells

**Figure 2:**
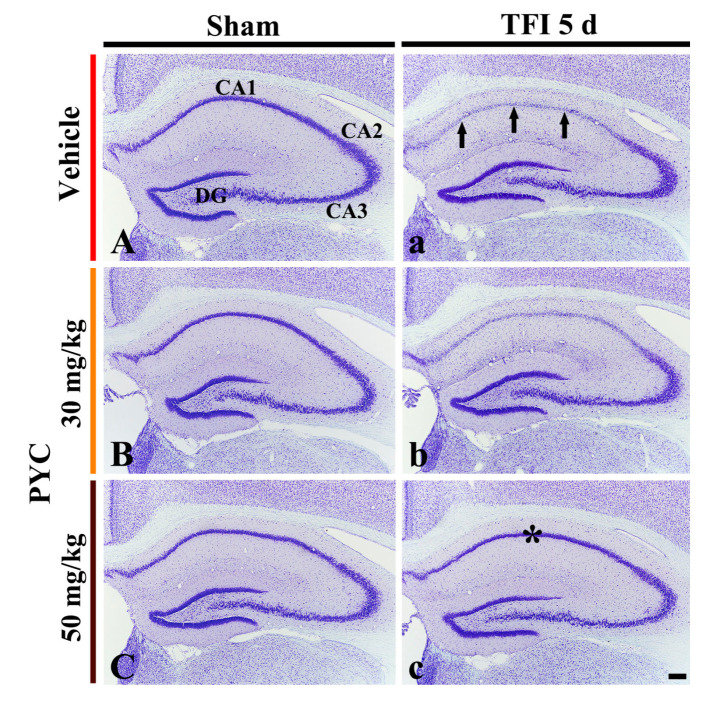
Cresyl Violet (CV) staining in the hippocampus of the vehicle/sham (**A**), vehicle/TFI (**a**), 30 mg/kg PYC/sham (**B**), 30 mg/kg PYC/TFI (**b**), 50 mg/kg PYC/sham (**C**), and 50 mg/kg PYC/TFI (**c**) groups at 5 days after TFI. In the vehicle/TFI group, CV stainability in the CA1 pyramidal cells (arrows) is very pale, whereas strong CV stainability is observed in the CA1 pyramidal cells (asterisk) of the 50 mg/kg PYC/TFI group. CA, cornu ammonis; DG, dentate gyrus. scale bar = 200 μm.

CV^+^ cells observed in the vehicle/sham group were easily shown in all hippocampal subareas (Figure 2A). In the 30, 40 and 50 mg/kg PYC/sham groups, no significant difference was found in the distribution and morphology of the CV^+^ cells in comparison with those found in the vehicle/sham group (Figure 2B,C) (data not shown at 40 mg/kg).

In the vehicle/TFI group, the stainability in the CV^+^ cells was distinctly decreased in pyramidal cells (neurons) located in the stratum pyramidale of the hippocampal CA1 area, not in the other subareas, at 5 days after TFI (Figure 2a). This result means that, in the CA1 area, pyramidal cells or neurons (called CA1 pyramidal cells) are damaged by TFI. In the 30 and 40 mg/kg PYC/TFI group, CV stainability in the CA1 pyramidal cells was not different from that found in the vehicle/TFI group (Figure 2b). However, the CV stainability in the CA1 pyramidal cells in the 50 mg/kg PYC/TFI group was similar to that shown in the vehicle/sham group (Figure 2c): this finding means that 40 mg/kg PYC pretreatment can protect CA1 pyramidal cells from TFI.

#### 3.2.2. NeuN^+^ Cells

**Figure 3:**
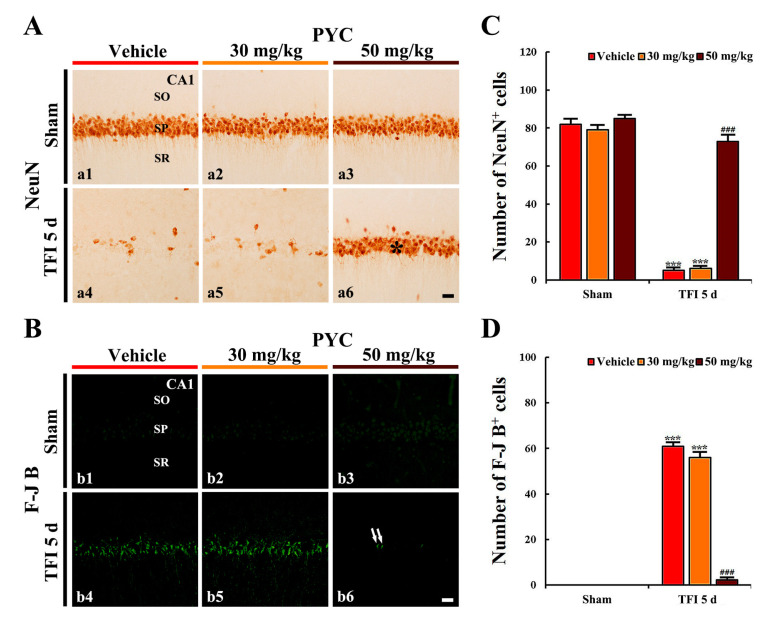
(**A**) Neuronal damage/death with neuronal nuclear antigen (NeuN) immunohistochemistry (a1–a6) and (**B**) Fluoro-Jade B (F-J B) histofluorescence staining (b1–b6) in the CA1 area of the vehicle/sham (a1,b1), 30 mg/kg PYC/sham (a2,b2), 50 mg/kg PYC/TFI (a3,b3), vehicle/TFI (a4,b4), 30 mg/kg PYC/TFI (a5,b5), and 50 mg/kg PYC/TFI (a6,b6) groups at 5 days after TFI. In the vehicle/TFI group, a few NeuN^+^ cells and many F-J B^+^ cells are observed in the stratum pyramidale (SP). However, in the 50 mg/kg PYC/TFI group, abundant NeuN^+^ (asterisk), and only a few F-J B^+^ CA1 pyramidal cells (arrows) are observed. SO, stratum oriens; SR, stratum radiatum. Scale bar = 10 μm. (C and D) The mean numbers of NeuN^+^ (**C**) and F-J B^+^ CA1 pyramidal cells (**D**). The bars indicate the means ± SEM (*n* = 7/group, *** *p* < 0.001 vs. each sham group, ^###^ *p* < 0.001 vs. vehicle/TFI group).

CA1 pyramidal cells in the vehicle/sham showed clear NeuN immunoreactivity (Figure 3A(a1)). In addition, in the 30, 40, and 50 mg/kg PYC/sham groups, NeuN immunoreactivity in the CA1 pyramidal cells was similar to that found in the vehicle/sham group (Figure 3A(a2,a3),C).

In the vehicle/TFI group, NeuN^+^ CA1 pyramidal cells were significantly decreased in numbers 5 days after TFI (*p* < 0.001; Figure 3A(a4)): the mean number of NeuN^+^ CA1 pyramidal cells was 5 cells/100 × 100 μm (Figure 2C). In the 30 and 40 mg/kg PYC/TFI group, the number of NeuN^+^ CA1 pyramidal neurons was not different in comparison with that in the vehicle/TFI group (Figure 3A(a5),C). In the 50 mg/kg PYC/TFI group, however, the number of NeuN^+^ CA1 pyramidal cells was significantly preserved (73 cells/100 × 100 μm) in comparison to that found in the vehicle/TFI group (*p* < 0.001) (Figure 3A(a6),C).

#### 3.2.3. F-J B^+^ Cells

F-J B^+^ cells were not detected in the CA1 area when we observed in all sham groups (Figure 3B(b1–b3)). In the vehicle/TFI group, however, many F-J B^+^ CA1 pyramidal ells (61 cells/100 × 100 μm) were found in the stratum pyramidale (Figure 3B(b4),D). In the 30 and 40 mg/kg PYC/TFI groups, the number and distribution pattern of F-J B^+^ CA1 pyramidal cells were not different from those found in the vehicle/TFI group (Figure 3B(b5),D). However, in the 50 mg/kg PYC/TFI group, few F-J B^+^ CA1 pyramidal neurons were detected (2 cells/250 × 250 μm) (Figure 3B(b6),D): this result means that only a few CA1 pyramidal cells die due to TFI. 

Based on results of CV staining, NeuN immunohistochemistry, and F-J B histofluorescence staining, we carried out following items to investigate the mechanisms of neuroprotection mediated by pretreatment with 50 mg/kg PYC against TFI in gerbil hippocampal CA1 area. 

### 3.3. Attenuated Oxidative Stress

In this study, pretreatment with 30 and 40 mg/kg of PYC, findings in DHE fluorescence, and OHG and 4HNE immunoreactivity were not significantly different from those found in the vehicle/TFI group. Therefore, the alterations were performed in the groups pretreated with 50 mg/kg PYC against TFI. 

#### 3.3.1. DHE Fluorescence

**Figure 4:**
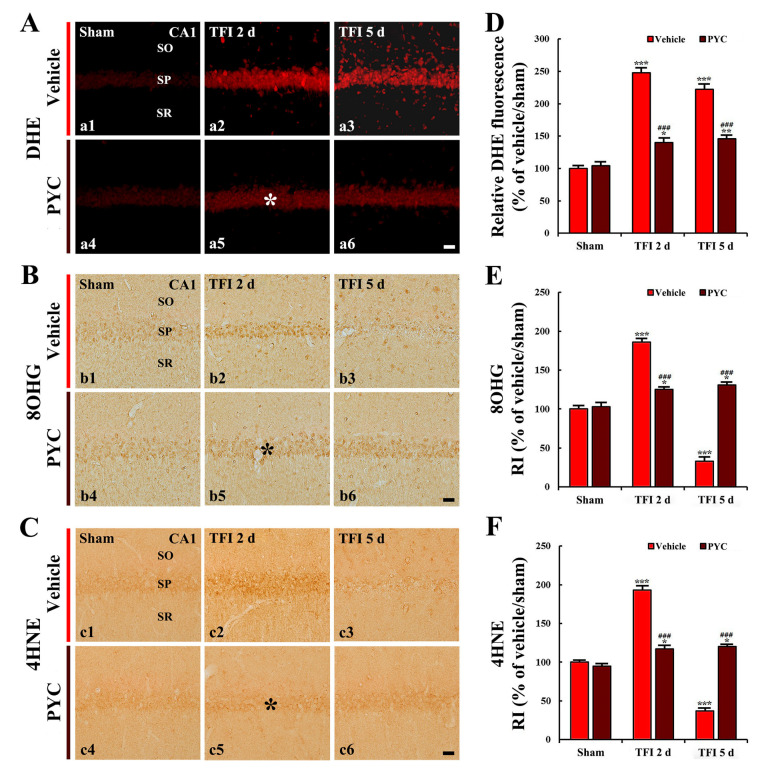
(**A**) Dihydroethidium (DHE) fluorescence staining (a1–a6), (**B**) 8-hydroxyguanine (8OHG) (b1–b6), and (**C**) 4-hydroxy-2-nonenal (4HNE) immunohistochemistry (c1–c6) in the CA1 area of the vehicle-treated groups (upper panels) and PYC-treated groups (lower panels) at sham (a1–c1,a4–c4), 2 days (a2–c2,a5–c5), and 5 days (a3–c3,a5–c5) after TFI. In the PYC/TFI group, DHE fluorescence intensity, 8OHG, and 4HNE immunoreactivities in CA1 pyramidal cells (asterisks) at 2 days after TFI are significantly lower than those in the vehicle/TFI group. Scale bar = 10 μm. (**D)**–(**F**) Quantitative analyses of DHE fluorescence intensity (**D**), 8OHG immunoreactivity (**E**), and 4HNE immunoreactivity (**F**) in CA1 pyramidal cells. The bars indicate the means ± SEM (*n* = 7/group; * *p* < 0.05, ** *p* < 0.01, *** *p* < 0.001 vs. each sham group, ^###^ *p* < 0.001 vs. corresponding vehicle-treated group).

DHE fluorescence intensity was weakly detected in the CA1 pyramidal neurons in the vehicle/sham group (Figure 4A(a1)). In the vehicle/TFI group, the intensity of DHE fluorescence in the CA1 pyramidal neurons was significantly enhanced 2 and 5 days after TFI (249% and 223%, respectively, of the vehicle/sham group; *p* < 0.001) (Figure 4A(a2,a3),D). Five days after TFI, in particular, DHE fluorescence intensity was strongly shown in non-pyramidal cells that were distributed in the other layers (strata oriens and radiatum) of the CA1 area (Figure 4A(a3)). 

In the PYC/sham group, the intensity of DHE fluorescence found in the CA1 pyramidal neurons was not different from that found in the vehicle/sham group (Figure 4A(a4),D). In the PYC/TFI group, DHE fluorescence intensity found in the CA1 pyramidal cells 2 days after TFI was significantly decreased (about 57% of the vehicle/TFI group) in comparison with that in the vehicle/TFI group (*p* < 0.001) (Figure 4A(a5),D), showing that the fluorescence intensity was not altered until 5 days after TFI (Figure 4A(a6),D). This finding means that the production of superoxide anion following TFI is attenuated by PYC.

#### 3.3.2. 8-OHG and 4HNE Immunoreactivities

Weak 8OHG and 4HNE immunoreactivity was shown in CA1 pyramidal cells in the vehicle/sham group (Figure 4B(b1),C(c1)). However, in the vehicle/TFI group, significantly enhanced 8OHG and 4HNE immunoreactivity (187% and 194%, respectively) was found in CA1 pyramidal neurons 2 days after TFI when compared with that found in the vehicle/sham group (*p* < 0.001; Figure 4B(b2),C(c2),E,F). Five days after TFI, 8OHG and 4HNE immunoreactivity was barely shown in the CA1 pyramidal neurons because the neurons died after TFI (Figure 4B(b3),C(c3),E,F). 

8OHG and 4HNE immunoreactivity found in CA1 pyramidal neurons of the PYC/sham group was not different from that found in the vehicle/sham group (Figure 4B(b4),C(c4),E,F). In the PYC/TFI group, significantly low 8OHG and 4HNE immunoreactivity (about 67% and 61% of the vehicle/TFI group, respectively) was found in the CA1 pyramidal neurons 2 days after TFI in comparison to that found in the vehicle/TFI group (*p* < 0.001; Figure 4B(b5),C(c5),E,F), and each immunoreactivity was not altered until 5 days after TFI (Figure 4B(b6),C(c6),E,F).

### 3.4. Increased Antioxidant Enzyme Immunoreactivities 

In this study, pretreatment with 30 and 40 mg/kg of PYC, findings in SODs and CAT immunoreactivity were not significantly different from those found in the vehicle/TFI group. Therefore, the alterations were examined in the groups treated with 50 mg/kg PYC. 

#### 3.4.1. SODs Immunoreactivity

**Figure 5:**
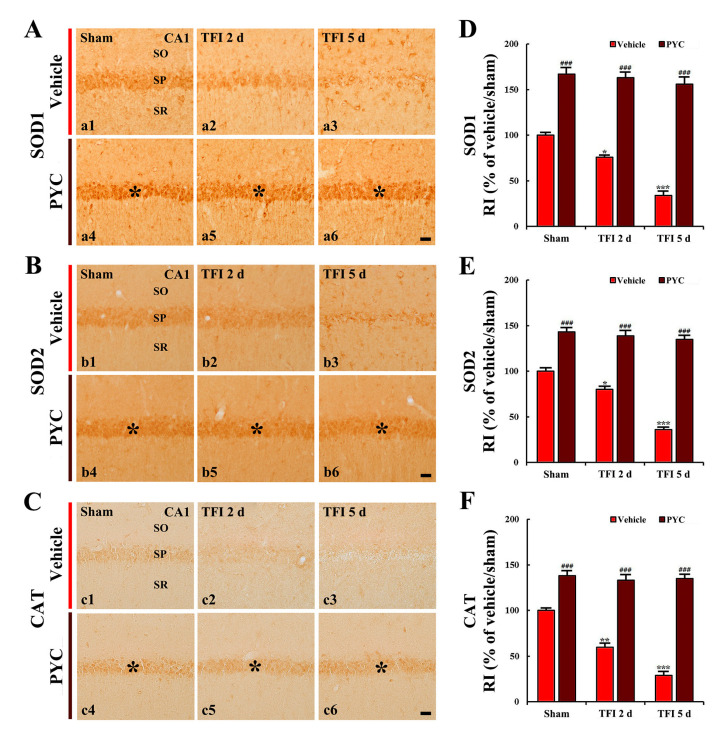
(**A**) SOD1 (a1–a6), (**B**) SOD2 (b1–b6), and (**C**) catalase (CAT) (c1–c6) immunohistochemistry in the CA1 area of the vehicle-treated group (upper panels) and PYC-treated group (lower panels) at sham (a1–c1, a4–c4), 2 days (a2–c2,a5–c5), and 5 days (a3–c3,a5–c5) after TFI. In the PYC/sham and PYC/TFI groups, SOD1, SOD2, and CAT immunoreactivity in CA1 pyramidal cells (asterisks) is significantly increased compared to that in the corresponding vehicle-treated group. Scale bar = 10 μm. (**D)**–(**F**) Quantitative analyses of SOD1 (**D**), SOD2 (**E**), and CAT (**F**) immunoreactivity in CA1 pyramidal cells. The bars indicate the means ± SEM (*n* = 7/group; * *p* < 0.05, ** *p* < 0.01, *** *p* < 0.001 vs. each sham group; ^###^ *p* < 0.001 vs. corresponding vehicle-treated group).

In the vehicle/sham group, SODs (SOD1 and SOD2) immunoreactivity was moderately detected in CA1 pyramidal neurons (Figure 5A(a1),B(b1)). In the vehicle/TFI group, the SOD1 and SOD2 immunoreactivity was significantly reduced 2 and 5 days TFI (76% and 80%, respectively, of the vehicle/sham group) (*p* < 0.05; Figure 5A(a2),B(b2),D,E). At this time after TFI, in particular, each immunoreactivity in the CA1 pyramidal cells became very low because the neurons were dead by TFI (Figure 5A(a3),B(b3),D,E). 

SOD1 and SOD2 immunoreactivity in the CA1 pyramidal neurons of the PYC/sham group was significantly high (168% and 144%, respectively, of the vehicle/sham group) when compared with that in the vehicle/sham group (*p* < 0.001; Figure 5A(a4),B(b4),D,E). In the PYC/TFI group, the increased SODs immunoreactivity was sustained until 5 days after TFI (Figure 5A(a5,a6),B(b5,b6),D,E).

#### 3.4.2. CAT Immunoreactivity

CAT immunoreactivity was weakly found in the CA1 pyramidal neurons in the vehicle/sham group (Figure 5C(c1)). However, In the vehicle/TFI group, the CAT immunoreactivity was significantly reduced 2 days after TFI by about 39% when compared with that found in the vehicle/sham group (*p* < 0.01; Figure 5C(c2),F), and CAT immunoreactivity at 5 days TFI was rarely observed because of the death of the CA1 pyramidal neurons by TFI (Figure 5C(c3),F).

In the PYC/sham group, CAT immunoreactivity found in the CA1 pyramidal cells was significantly increased (139% of the vehicle/sham group) in comparison with that found in the vehicle/sham group (*p* < 0.001; Figure 5C(c4),F). In the PYC/TFI group, the enhanced CAT immunoreactivity was sustained in the CA1 pyramidal cells until 5 days post-TFI (Figure 5C(c5,c6),F).

### 3.5. SA Effect on Oxidative Stress

**Figure 6:**
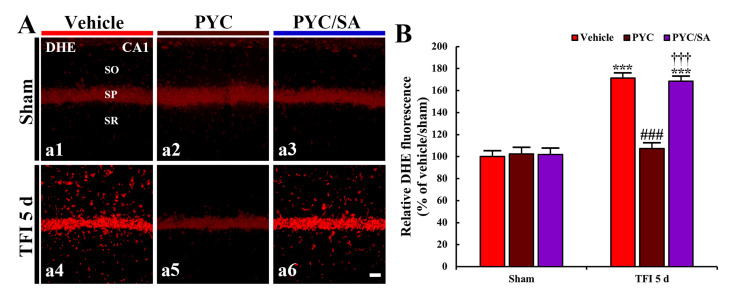
(**A**) DHE fluorescence (a1–a6) in the CA1 area of the vehicle-treated (left column), PYC-treated (middle column) and PYC/sodium azide (SA)-treated (right column) group at 5 days after sham (upper panels) and TFI (lower panels). In the PYC/TFI group, DHE fluorescence intensity is significantly weaker than that in the vehicle/TFI group. However, in the PYC/SA/TFI group, DHE fluorescence intensity is similar to that in the vehicle/TFI group. Scale bar = 10 μm. (**B**) Quantitative analysis of DHE fluorescence intensity in CA1 pyramidal cells. The bars indicate the means ± SEM (*n* = 7/group; *** *p* < 0.001 vs. each sham group, ^###^ *p* < 0.001 vs. corresponding vehicle-treated group, ^†††^; *p* < 0.001 vs. corresponding PYC-treated group).

Pyramidal cells in all sham groups showed weak DHE fluorescence intensity (Figure 6A(a1–a3)). DHE fluorescence intensity, in the vehicle/TFI group, was significantly enhanced (171% of the vehicle/sham group, *p* < 0.001) compared to that found in the vehicle sham group (Figure 6A(a4),B). In the PYC/TFI group, a significant reduction in DHE fluorescence was detected (about 63% of the vehicle/TFI group, *p* < 0.001) compared to that found in the vehicle/TFI group (Figure 6A(a5),B). However, in the PYC/SA/TFI group, DHE fluorescence intensity was not different form that found in the vehicle/TFI group (Figure 6A(a6),B).

### 3.6. Disappearance of Neuroprotection by SA

#### 3.6.1. NeuN^+^ Cells

**Figure 7:**
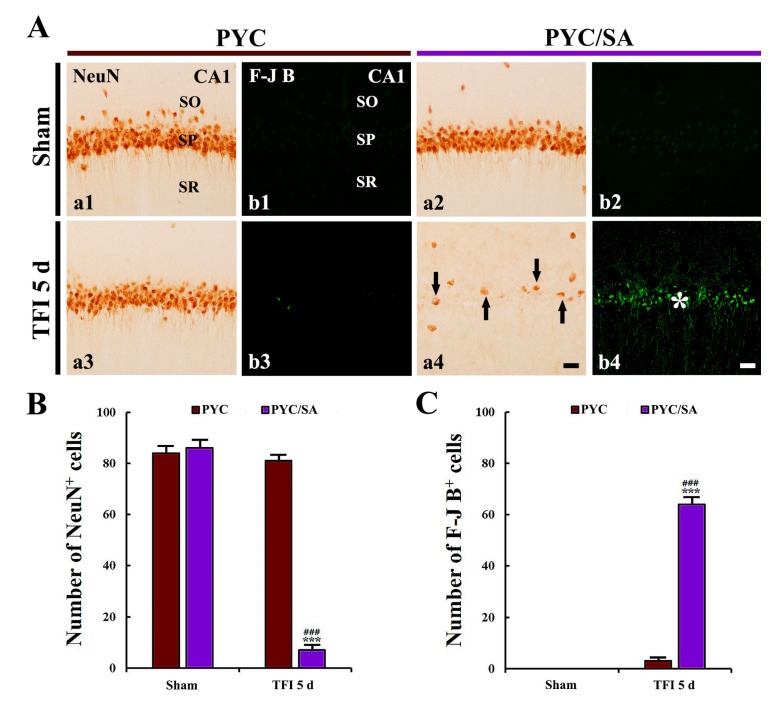
(**A**) NeuN immunohistochemistry (a1–a4) and F-J B histofluorescence staining (b1–b4) in the CA1 area of the PYC/sham (a1,b1), PYC/SA/sham (a2,b2), PYC/TFI (a3,b3), and PYC/SA/TFI (a4,b4) groups at 5 days after TFI. In the PYC/SA/TFI group, a few NeuN^+^ CA1 pyramidal cells (arrows) and many F-J B^+^ CA1 pyramidal cells (asterisk) are observed. SO, stratum oriens; SP, stratum pyramidale; SR, stratum radiatum. Scale bar = 10 μm. (**B**,**C**) The mean numbers of NeuN^+^ (**B**) and F-J B^+^ cells (**C**) in the CA1 area. The bars indicate the means ± SEM (*n* = 7/group, *** *p* < 0.001 vs. PYC/sham group, ^###^ *p* < 0.001 vs. PYC/TFI group).

In the PYC/SA/sham group, numbers and distribution of NeuN^+^ CA1 pyramidal neurons were not different from those found in the PYC/sham group (Figure 7A(a1,a2),B). 

In the PYC/TFI group, abundant NeuN^+^ CA1 pyramidal cells (81 cells/100 × 100 μm) were observed;, whereas, in the PYC/SA/TFI group, NeuN^+^ CA1 pyramidal neurons were significantly reduced (7 cells/100 × 100 μm) at 5 days post-TFI in comparison with those found in the PYC/TFI group (*p* < 0.001; Figure 7A(a4,b4),B,C).

#### 3.6.2. F-J B^+^ Cells

F-J B^+^ pyramidal cells were not found in the CA1 area observed in the PYC/SA/sham group (Figure 7A(b1,b2),C).

In the PYC/TFI group, a few F-J B^+^ pyramidal cells (3 cells/100 × 100 μm) were detected at 5 days post-TFI (Figure 7A(a3,b3),B,C). However, in the PYC/SA/TFI group, F-J B^+^ pyramidal neurons were significantly increased (64 cells/100 × 100 μm) at 5 days after TFI in comparison with those found in the PYC/TFI group (*p* < 0.001; Figure 7A(a4,b4),B,C).

## 4. Discussion

Historically, medicinal plants have been proven their value as therapeutic sources for neurological disorders, including cerebral ischemia (ischemic stroke) [34,35,36]. In our current study, we evaluated the neuroprotective effects of pretreated PYC originating from the maritime pine bark in the CA1 area of gerbil hippocampus following TFI using histochemistry with CV, immunohistochemistry with NeuN, and histofluorescence with F-J B. It was reported that PYC protected neuronal pheochromocytoma (PC)12 cells from hydrogen peroxide-induced injury in vitro [37]. In addition, a recent study reported that pretreated with 30 mg/kg PYC protected cerebral cortical neurons against transient focal brain ischemia induced by occlusion of middle cerebral artery (OMCA) in rats [16]. Based on the previous studies, we set dosages of PYC from 30 to 50 mg/kg with 10 mg/kg interval of the dosage and found that treatment with 50 mg/kg PYC effectively protected pyramidal cells located in the stratum pyramidale in the CA1 area against TFI. This is the first study demonstrating neuroprotective effects of PYC against TFI, which is different in the damaged brain areas and the process of neuronal or parenchymal damage from those in transient focal brain ischemia. Namely, transient focal brain ischemia induced by OMCA in a rat is induced for at least 1 h, and the ischemic damage represents infarction (necrosis of tissue), showing that the damage evokes motor dysfunction due to ischemic damage in the striatum and cerebral cortex [25,38]. In contrast, 5-min TFI evokes selective neuronal loss/death in the CA1 region of gerbil hippocampus and develops memory deficits [17,18,19,20]. Namely, 5-min TFI in the gerbil brings death or loss of pyramidal neurons located in the CA1 area from 4–5 days after TFI [39,40].

It has been reported that the hippocampus is very associated with learning and memory function [41,42]. This function has been assessed by well-established behavioral tests: (1) PAT for the evaluation of short-term and/or long-term memory function [43] and (2) 8-arm radial maze test for the measure of spatial memory function [44]. It has been reported that deficits in learning and memory are developed in gerbils after 5 min TFI, and the deficits result from the death of CA1 pyramidal neurons induced by TFI [19,45]. In addition, our current cognitive tests showed that learning and memory by the PAT, and spatial memory observed by 8-arm radial maze test was apparently reduced following TFI. This outcome was similar to that found in the precedent studies. However, in our study, the pretreatment with 50 mg/kg PYC showed that the cognitive deficits were attenuated. This amelioration of the cognitive deficits must be resulted from the impact that the pretreatment with 50 mg/kg PYC prevented the loss of the CA1 pyramidal cells following TFI. The neuroprotection was identified by histochemistry with CV, immunohistochemistry with NeuN, and histofluorescence with F-J B.

Accumulating evidence has demonstrated that cerebral ischemia provokes massive production of ROS which result in oxidative stress-mediated damage to cellular components, such as DNA, ribonucleic acid (RNA), proteins, and lipids, ultimately leading to neuronal death in ischemic brains [25,46]. Studies with animal models of cerebral ischemia have shown that pharmacological inhibition of cerebral ischemia-induced oxidative stress strongly contributes to neuroprotection against ischemic brain injury [5,37,47]. In our current study, the immunoreactivities of DHE (a probe of superoxide anion), 8OHG (a marker of RNA oxidation), and 4HNE (a marker of lipid peroxidation) were significantly increased in the CA1 pyramidal cells after TFI, but they were apparently decreased in the ischemic CA1 pyramidal neurons by pretreatment with PYC (50 mg/kg). This result indicates that TFI-induced oxidative stress in the CA1 pyramidal cells is attenuated by PYC pretreatment, which is closely associated with PYC-mediated neuroprotection against TFI.

It is well accepted that antioxidant enzymes, such SODs and CAT as free radical scavengers play a prominent role in neuroprotection against ischemic injury in brains. For example, ischemic brain injuries following transient global or focal ischemia in brains are apparently ameliorated in genetically engineered mouse and rat models overexpressing SOD1, SOD2, or CAT [48,49,50]. Moreover, it has been reported that enhanced levels of SOD and CAT by pretreatment with some plant extracts are closely involved in neuroprotection against ischemic injury following transient global ischemia in brains of rats and gerbils [51,52,53]. In our current study, immunoreactivities of SOD1, SOD2, and CAT in the CA1 pyramidal cells of the PYC/sham group were significantly high in comparison with those found in the vehicle/sham group, and the increased immunoreactivities in the CA1 pyramidal cells by PYC pretreatment were sustained until 5 days after TFI. This finding meant that the survival of the CA1 pyramidal neurons from TFI depends on PYC pretreatment.

SA, a non-colored crystalline solid, is well known to act as an inhibitor of endogenous antioxidant enzymes, including SODs (SOD1 and 2) and CAT [33,54,55]. Since the SA shows the inhibitory activity of such enzymes, it has been used in order to give oxidative stress. For example, Somade et al. (2016) utilized a rat model of extra-hepatic oxidative stress induced by SA [33]. In addition, Gao et al. (2018) triggered oxidative stress in rat PC12 cells through administration of SA [56]. Based on these precedent studies, we currently administered SA at 10 min followed by PYC treatment. Interestingly, oxidative stress was not attenuated by PYC following SA administration. Eventually, this might abolish the PYC-mediated neuroprotection against TFI-induced injury. Thus, these results strongly indicate that the upregulation of SODs and CAT in the CA1 pyramidal cells by PYC pretreatment provides survival of the CA1 pyramidal cells from TFI.

In summary, our current study clearly showed that pretreated PYC ameliorated declines in working and memory function induced by TFI effectively protected hippocampal CA1 pyramidal cells against ischemic injury induced by TFI, showing that the neuroprotective effect closely depended on the antioxidant role of PYC against TFI-induced oxidative stress. Taken together, our findings provide information on neuroprotective action of PYC against ischemic stroke, as a health functional food to prevent ischemic brain injury.

## Figures and Tables

**Table 1:** Composition of Pycnogenol^®^ (PYC).

**Phenolic Compounds**	Phenolic acids	Benzoic acid	*p*-Hydroxybenzoic acid
			protocatechic acid
			Vanilic acid
			Gallic acid
		Cinnamic acid	
		*p*-Cumaric acid	
		Caffeic acid	
		Ferulic acid	
	Catechin	Procyanidin	Major component (70 ± 5% of standardized procyanidins)
		Epicatechin	
	Taxifolin	Taxifolin glucoside	
**Inorganic Ions**	calcium, potassium, iron, manganese, zinc, copper, selenium		
